# Supplementary material for: Genomic characterization and phylogenetic analysis of Salmonella enterica serovar Javiana
Source: PeerJ. 2020 Nov 20;8:e10256. doi: 10.7717/peerj.10256 (PMC7682435; doi:10.7717/peerj.10256)
Supplement: Figure S2 — Minimal spanning trees were constructed from cgMLST data using GrapeTree on Enterobase with the improved minimal spanning tree algorithm (MSTree V2). Separate trees were constructed for each (A) Salmonella ser. Javiana, (B) ser. Derby, (C) ser. Kentucky, (D) ser. Mississippi, (E) ser. Montevideo, (F) ser. Newport, (G) ser. Saintpaul, and (H) ser. Senftenberg. Nodes are color coded by ceBG designations (see legend in each panel) and ceBG designations associated with each serovar are in red boxes. Branch lengths (representing cgMLST allelic differences) are shown in red above branches between ceBG clusters. [file peerj-08-10256-s002.pdf]

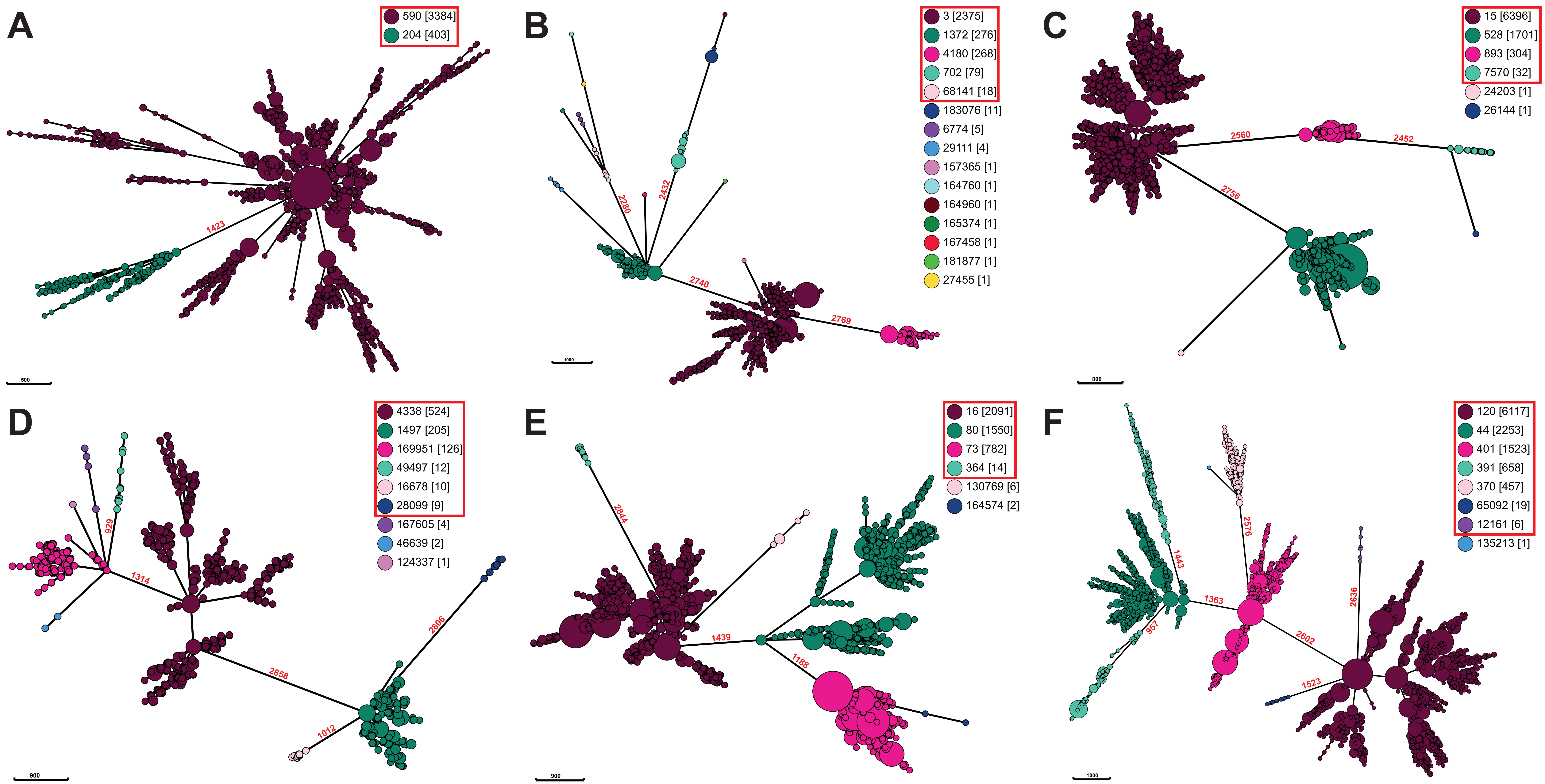

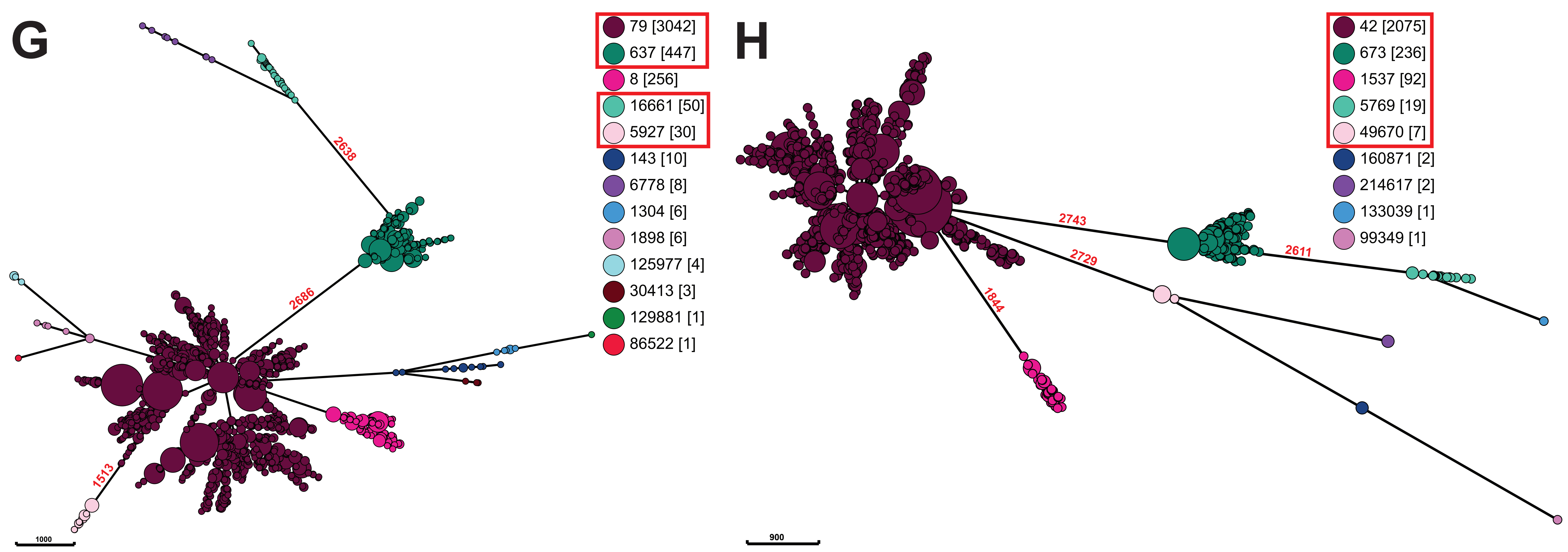

Figure S2. Minimal Spanning Trees of *Salmonella* ser. Javiana and Polyphyletic Serovar Strains.

Minimal spanning trees were constructed from cgMLST data using GrapeTree on Enterobase with the improved minimal spanning tree algorithm (MSTree V2). Separate trees were constructed for each (A) *Salmonella* ser. Javiana, (B) ser. Derby, (C) ser. Kentucky, (D) ser. Mississippi, (E) ser. Montevideo, (F) ser. Newport, (G) ser. Saintpaul, and (H) ser. Senftenberg. Nodes are color coded by ceBG designations (see legend in each panel) and ceBG designations associated with each serovar (as reported in the EnteroBase documentation (EnteroBase Team, 2018)) are in red boxes. Branch lengths (representing cgMLST allelic differences) are shown in red above branches between ceBG clusters.
