# Supplementary figures and images for: Genomic characterization and phylogenetic analysis of Salmonella enterica serovar Javiana
Source: PeerJ. 2020 Nov 20;8:e10256. doi: 10.7717/peerj.10256 (PMC7682435; doi:10.7717/peerj.10256)

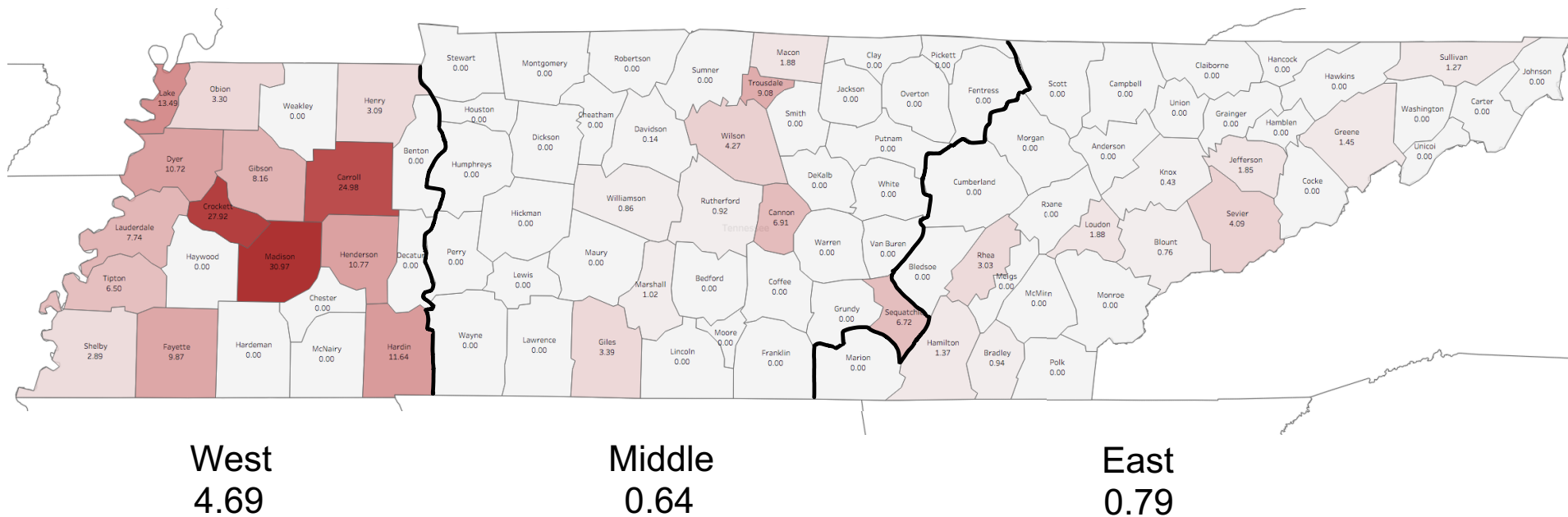

**Figure S3.** Map of Incidence Rates

Supplement: Figure S3 [file peerj-08-10256-s003.pdf]
