# Supplementary material for: Genomic characterization and phylogenetic analysis of Salmonella enterica serovar Javiana
Source: PeerJ. 2020 Nov 20;8:e10256. doi: 10.7717/peerj.10256 (PMC7682435; doi:10.7717/peerj.10256)
Supplement: Figure S1 — Tree was constructed based on core SNPs determined by KSNP3 (Gardner, Slezak & Hall, 2015). The optimal tree with the sum of branch length of 31,777.6 is shown. The percentage of replicate trees in which the associated taxa clustered together in the bootstrap test are represented by branch color (maximum as green, midpoint as yellow, and minimum as red). The tree is drawn to scale, with branch lengths (above branches) representing the number of base differences at core SNP positions per isolate (SNP distance). The analysis involved 161 isolates and 30,657 total SNP positions. The three major clades are labeled. HC900 (ceBG) clusters are indicated (590 is not shaded and 204 is shaded in gray). TN isolates belonging to TN clades I, II, and III from our original analysis (Fig. 1) are highlighted in purple, green, and blue, respectively. Metadata, including HC100 cluster designations, country, region/state, and collection year are listed to the right of node labels. [file peerj-08-10256-s001.pdf]

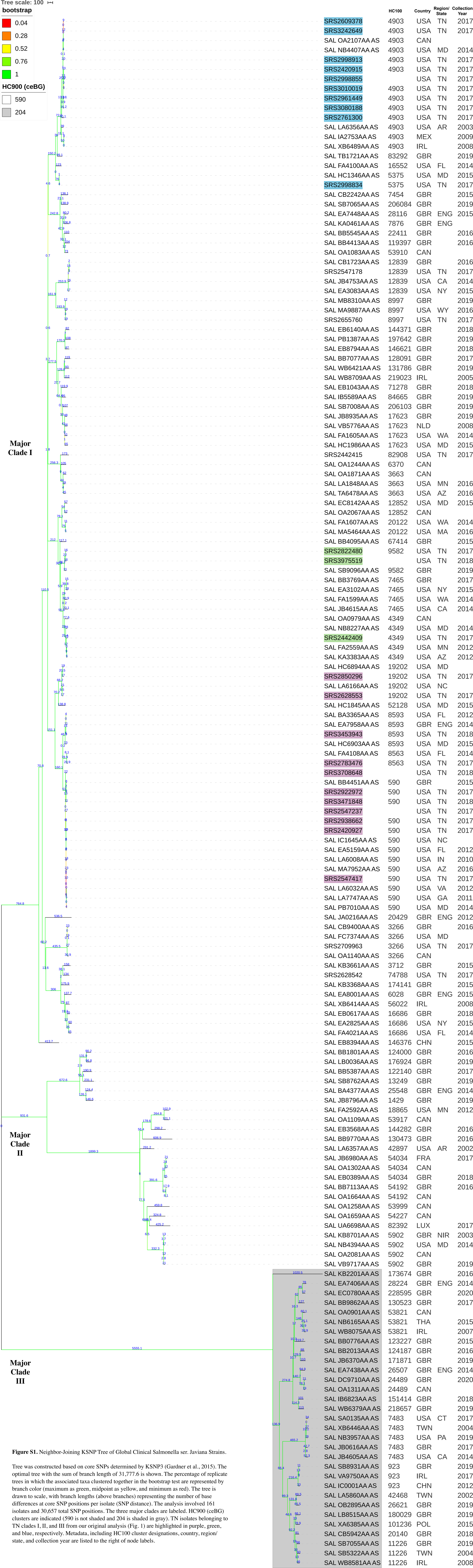

Isolate	HC100	Country	Region/ State	Collection Year
SRS2609378	4903	USA	TN	2017
SRS3242649	4903	USA	TN	2017
SAL OA2107AA AS	4903	CAN		
SAL NB4407AA AS	4903	USA	MD	2014
SRS2998913	4903	USA	TN	2017
SRS2420915	4903	USA	TN	2017
SRS2998855		USA	TN	2017
SRS3010019	4903	USA	TN	2017
SRS2961449	4903	USA	TN	2017
SRS3080188	4903	USA	TN	2017
SRS2761300	4903	USA	TN	2017
SAL LA6356AA AS	4903	USA	AR	2003
SAL IA2753AA AS	4903	MEX		2009
SAL XB6489AA AS	4903	IRL		2008
SAL TB1721AA AS	83292	GBR		2019
SAL FA4100AA AS	16552	USA	FL	2014
SAL HC1346AA AS	5375	USA	MD	2015
SRS2998834	5375	USA	TN	2017
SAL CB2242AA AS	7454	GBR		2015
SAL SB7065AA AS	206084	GBR		2019
SAL EA7448AA AS	28116	GBR	ENG	2015
SAL KA0461AA AS	7876	GBR	ENG	
SAL BB5545AA AS	22411	GBR		2016
SAL BB4413AA AS	119397	GBR		2016
SAL OA1083AA AS	53910	CAN		
SAL CB1723AA AS	12839	GBR		2016
SRS2547178	12839	USA	TN	2017
SAL JB4753AA AS	12839	USA	CA	2014
SAL EA3083AA AS	12839	USA	NY	2015
SAL MB8310AA AS	8997	GBR		2019
SAL MA9887AA AS	8997	USA	WY	2016
SRS2655760	8997	USA	TN	2017
SAL EB6140AA AS	144371	GBR		2018
SAL PB1387AA AS	197642	GBR		2019
SAL EB8794AA AS	146621	GBR		2018
SAL BB7077AA AS	128091	GBR		2017
SAL WB6421AA AS	131786	GBR		2019
SAL WB8709AA AS	219023	IRL		2005
SAL EB1043AA AS	71278	GBR		2018
SAL IB5589AA AS	84665	GBR		2019
SAL SB7008AA AS	206103	GBR		2019
SAL JB8935AA AS	17623	GBR		2019
SAL VB5776AA AS	17623	NLD		2008
SAL FA1605AA AS	17623	USA	WA	2014
SAL HC1986AA AS	17623	USA	MD	2015
SRS2442415	82908	USA	TN	2017
SAL OA1244AA AS	6370	CAN		
SAL OA1871AA AS	3663	CAN		
SAL LA1848AA AS	3663	USA	MN	2016
SAL TA6478AA AS	3663	USA	AZ	2016
SAL EC8142AA AS	12852	USA	MD	2015
SAL OA2067AA AS	12852	CAN		
SAL FA1607AA AS	20122	USA	WA	2014
SAL MA5464AA AS	20122	USA	MA	2016
SAL BB4095AA AS	67414	GBR		2015
SRS2822480	9582	USA	TN	2017
SRS3975519		USA	TN	2018
SAL SB9096AA AS	9582	GBR		2019
SAL BB3769AA AS	7465	GBR		2017
SAL EA3102AA AS	7465	USA	NY	2015
SAL FA1599AA AS	7465	USA	WA	2014
SAL JB4615AA AS	7465	USA	CA	2014
SAL OA0979AA AS	4349	CAN		
SAL NB8227AA AS	4349	USA	MD	2014
SRS2442409	4349	USA	TN	2017
SAL FA2559AA AS	4349	USA	MN	2012
SAL KA3383AA AS	4349	USA	AZ	2012
SAL HC6894AA AS	19202	USA	MD	
SRS2850296	19202	USA	TN	2017
SAL LA6166AA AS	19202	USA	NC	
SRS2628553	19202	USA	TN	2017
SAL HC1845AA AS	52128	USA	MD	2015
SAL BA3365AA AS	8593	USA	FL	2012
SAL EA7958AA AS	8593	GBR	ENG	2014
SRS3453943	8593	USA	TN	2018
SAL HC6903AA AS	8593	USA	MD	2015
SAL FA4108AA AS	8563	USA	FL	2014
SRS2783476	8563	USA	TN	2017
SRS3708648		USA	TN	2018
SAL BB4451AA AS	590	GBR		2015
SRS2922972	590	USA	TN	2017
SRS3471848	590	USA	TN	2018
SRS2547237		USA	TN	2017
SRS2938662	590	USA	TN	2017
SRS2420927	590	USA	TN	2017
SAL IC1645AA AS	590	USA	NC	
SAL EA5159AA AS	590	USA	FL	2012
SAL LA6008AA AS	590	USA	IN	2010
SAL MA7952AA AS	590	USA	AZ	2016
SRS2547417	590	USA	TN	2017
SAL LA6032AA AS	590	USA	VA	2012
SAL LA7747AA AS	590	USA	GA	2011
SAL PB7010AA AS	590	USA	MD	2014
SAL JA0216AA AS	20429	GBR	ENG	2012
SAL CB9400AA AS	3266	GBR		2016
SAL FC7374AA AS	3266	USA	MD	
SRS2709963	3266	USA	TN	2017
SAL OA1140AA AS	3266	CAN		
SAL KB3661AA AS	3712	GBR		2015
SRS2628542	74788	USA	TN	2017
SAL KB3368AA AS	174141	GBR		2015
SAL EA8001AA AS	6028	GBR	ENG	2015
SAL XB6414AA AS	56022	IRL		2008
SAL EB0617AA AS	16686	GBR		2018
SAL EA2825AA AS	16686	USA	NY	2015
SAL FA4021AA AS	16686	USA	FL	2014
SAL EB8394AA AS	146376	CHN		2015
SAL BB1801AA AS	124000	GBR		2016
SAL LB0036AA AS	176924	GBR		2019
SAL BB5387AA AS	122140	GBR		2017
SAL SB8762AA AS	13249	GBR		2019
SAL BA4377AA AS	25548	GBR	ENG	2014
SAL JB8796AA AS	1429	GBR		2019
SAL FA2592AA AS	18865	USA	MN	2012
SAL OA1109AA AS	53917	CAN		
SAL EB3568AA AS	144282	GBR		2016
SAL BB9770AA AS	130473	GBR		2016
SAL LA6357AA AS	42897	USA	AR	2002
SAL JB6980AA AS	54034	FRA		2017
SAL OA1302AA AS	54034	CAN		
SAL EB0389AA AS	54034	GBR		2018
SAL BB7113AA AS	54192	GBR		2016
SAL OA1664AA AS	54192	CAN		
SAL OA1258AA AS	53999	CAN		
SAL OA1659AA AS	54227	CAN		
SAL UA6698AA AS	82392	LUX		2017
SAL KB8701AA AS	5902	GBR	NIR	2003
SAL NB4394AA AS	5902	USA	MD	2014
SAL OA2081AA AS	5902	CAN		
SAL VB9717AA AS	5902	GBR		2019
SAL KB2201AA AS	173674	GBR		2016
SAL EA7406AA AS	28224	GBR	ENG	2014
SAL EC0780AA AS	228595	GBR		2020
SAL BB9862AA AS	130523	GBR		2017
SAL OA0901AA AS	53821	CAN		
SAL NB6165AA AS	53821	THA		2015
SAL WB8075AA AS	53821	IRL		2007
SAL BB0776AA AS	123227	GBR		2015
SAL BB2013AA AS	124187	GBR		2016
SAL JB6370AA AS	171871	GBR		2019
SAL EA7438AA AS	26507	GBR	ENG	2014
SAL DC9710AA AS	24489	GBR		2020
SAL OA1311AA AS	24489	CAN		
SAL IB6823AA AS	151414	GBR		2018
SAL WB6379AA AS	218657	GBR		2019
SAL SA0135AA AS	7483	USA	CT	2017
SAL XB6446AA AS	7483	TWN		2004
SAL NB3957AA AS	7483	USA	PA	2019
SAL JB0616AA AS	7483	GBR		2017
SAL JB4605AA AS	7483	USA	CA	2014
SAL SB8931AA AS	923	GBR		2019
SAL VA9750AA AS	923	IRL		2017
SAL IC0001AA AS	923	CHN		2012
SAL LA5860AA AS	42468	TWN		2002
SAL OB2895AA AS	26621	GBR		2019
SAL LB8515AA AS	180029	GBR		2019
SAL XA6385AA AS	101236	POL		2015
SAL CB5942AA AS	20140	GBR		2018
SAL SB7055AA AS	11226	GBR		2019
SAL SB5322AA AS	11226	TWN		2004
SAL WB8581AA AS	11226	IRL		2008

Figure S1. Neighbor-Joining KSNP Tree of Global Clinical Salmonella ser. Javiana Strains.

Tree was constructed based on core SNPs determined by KSNP3 (Gardner et al., 2015). The optimal tree with the sum of branch length of 31,777.6 is shown. The percentage of replicate trees in which the associated taxa clustered together in the bootstrap test are represented by branch color (maximum as green, midpoint as yellow, and minimum as red). The tree is drawn to scale, with branch lengths (above branches) representing the number of base differences at core SNP positions per isolate (SNP distance). The analysis involved 161 isolates and 30,657 total SNP positions. The three major clades are labeled. HC900 (ceBG) clusters are indicated (590 is not shaded and 204 is shaded in gray). TN isolates belonging to TN clades I, II, and III from our original analysis (Fig. 1) are highlighted in purple, green, and blue, respectively. Metadata, including HC100 cluster designations, country, region/ state, and collection year are listed to the right of node labels.
