# Supplementary material for: Genomic characterization and phylogenetic analysis of Salmonella enterica serovar Javiana
Source: PeerJ. 2020 Nov 20;8:e10256. doi: 10.7717/peerj.10256 (PMC7682435; doi:10.7717/peerj.10256)
Supplement: Figure S4 — Circular representation of the 87.5 kb found in TN clade II isolate SRS2822480. Genes in green signify over-represented Clade II genes and their corresponding Prokka annotations. RASTtk annotations that differ from the Prokka annotations are in gray. Figure was created using Geneious Prime 2019.1.1 ( https://www.geneious.com ). [file peerj-08-10256-s004.pdf]

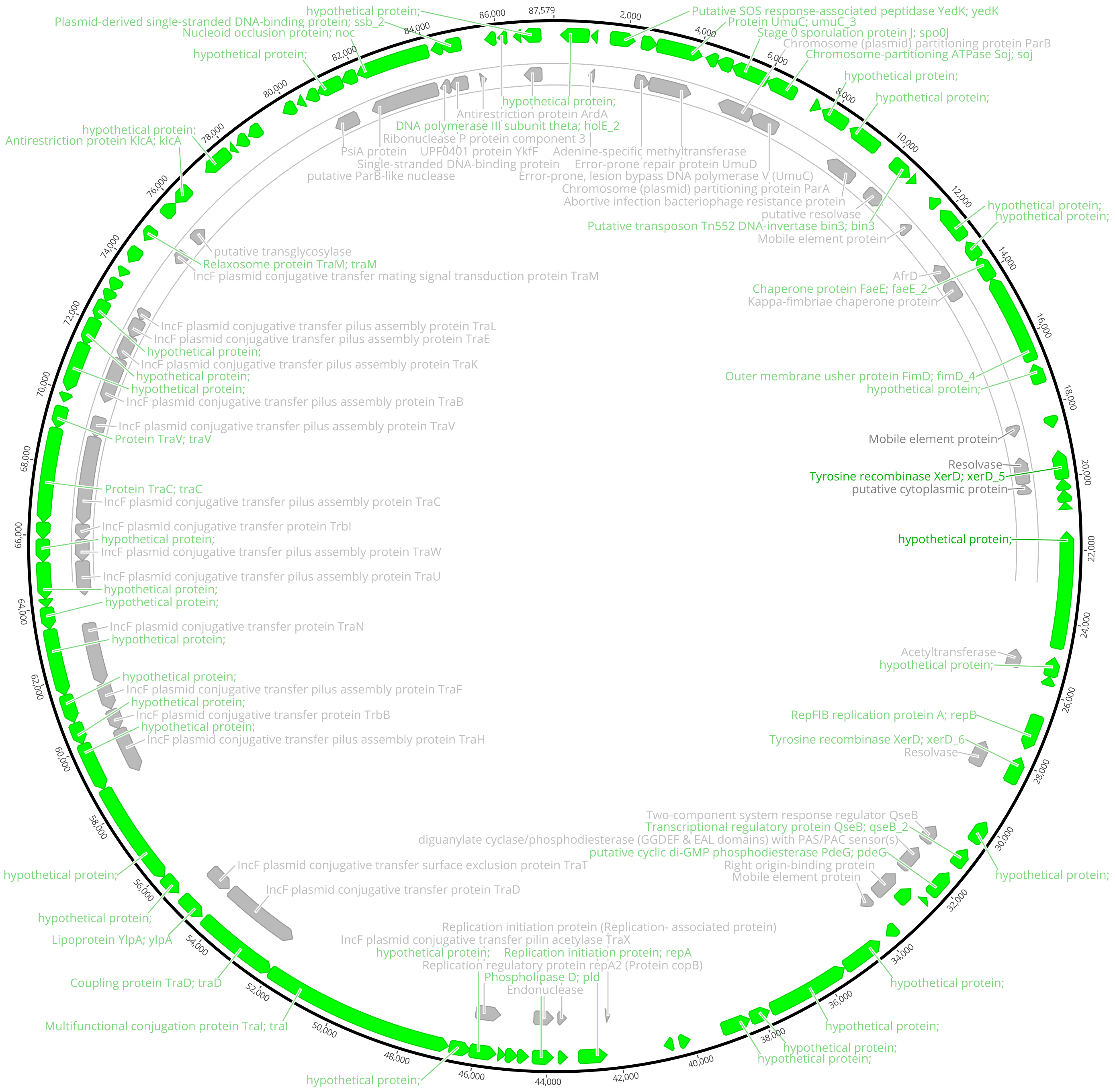

Figure S4. Clade II Plasmid
Circular representation of the 87.5 kb found in TN clade II isolate SRS2822480. Genes in green signify over-represented Clade II genes and their corresponding Prokka annotations. RASTtk annotations that differ from the Prokka annotations are in gray. Figure was created using Geneious Prime 2019.1.1 (https://www.geneious.com).
